# Deprotonation-Induced Conductivity Shift of Polyethylenedioxythiophenes in Aqueous Solutions: The Effects of Side-Chain Length and Polymer Composition

**DOI:** 10.3390/polym11040659

**Published:** 2019-04-10

**Authors:** Hailemichael Ayalew, Tian-lin Wang, Hsiao-hua Yu

**Affiliations:** 1 Smart Organic Materials Laboratory, Institute of Chemistry, Academia Sinica, 128 Sec. 2, Academia Road, Nankang, Taipei 11529, Taiwan; gishenm@gmail.com (H.A.); begood0325@gmail.com (T.-l.W.); 2 Taiwan International Graduate Program (TIGP), Sustainable Chemical Science and Technology (SCST), Academia Sinica, Taipei 11529, Taiwan; 3 Department of Applied Chemistry, National Chiao Tung University, Hsinchu 300, Taiwan

**Keywords:** poly(3,4-ethylenedioxythiophene)s, drain current measurement, conductivity shift, sensor, self-doping

## Abstract

Deprotonation-induced conductivity shift of poly(3,4-ethylenedixoythiophene)s (PEDOTs) in aqueous solutions is a promising platform for chemical or biological sensor due to its large signal output and minimum effect from material morphology. Carboxylic acid group functionalized poly(C_n_-EDOT-COOH)s are synthesized and electrodeposited on microelectrodes. The microelectrodes are utilized to study the effect of carboxylic acid side-chain length on the conductivity curve profiles in aqueous buffer with different pH. The conductivity shifts due to the buffer pH are effected by the length of the carboxylic acid side-chains. The shifts can be explained by the carboxylic acid dissociation property (p*K*_a_) at the solid–liquid interface, self-doping effect, and effective conjugation length. Conductivity profiles of poly(EDOT-OH-*co*-C_2_-EDOT-COOH) copolymers are also studied. The shifts show linear relationship with the feed monomer composition used in electrochemical polymerization.

## 1. Introduction

Nowadays, human health and environmental monitoring has become a critical issue due to ever-growing diseases and pollution concerns. This ensures a need for improvement or continuous development of sensory devices which are portable, cheaper, sensitive, and biocompatible, meaning they can respond faster to changes and chemical/biological species [1]. In this regard, electrically conducting polymers (ECPs) proved their widespread applications in the manufacturing of potentiometric [2], conductometric [3], or amperometric [4] chemical-/bio-sensors in the past decades [5,6]. This is because of their high sensitivity by varying their conductivity profile as a result of small perturbations in their oxidation state, chemical structure, and solid-state ordering [7,8]. Among the well-known conducting polymers, poly(3,4-ethylenedioxythiophene) (PEDOT) and its derivatives are the most studied ones due to their superior properties, including high electrical conductivity, stability in air at the oxidized state, versatile side-chain functionalizations, and ease of deposition on different substrates from aqueous and non-aqueous solutions [9,10,11,12,13].

From PEDOT derivatives, carboxylic acid-functionalized PEDOTs (poly(EDOT-COOH)) are by far the most studied derivatives for several applications including chemical-/bio-sensors [14,15,16] and nanobiointerfaces [17,18]. This is because functionalizing EDOT with carboxyl group gives a myriad of advantages, such as increasing PEDOT solubility in aqueous solvents, giving the self-doping nature of the polymer during oxidation/reduction process, enabling tuning of the polymer property by grafting of the carboxyl group with various functional groups, and enhancing the bioconjugation of PEDOT for immobilization of biomolecules for different purposes [14]. In addition, as reported by our group, poly(EDOT-COOH) polymer film is biocompatible, has low intrinsic cytotoxicity, and no inflammatory response for implantation, and we suggested that it is an ideal polymer for the preparation of biosensors [17]. Furthermore, the versatility of poly(EDOT-COOH) polymers in the development of chemical-/bio-sensors is demonstrated by our group and others too. For example, in one of our study, we reported a poly(C_2_-EDOT-COOH) polymer based sensor for label free detection of protein [15]. In other study, we prepared a poly(EDOT-OH-*co*-C_2_-EDOT-COOH) copolymer by electrochemical copolymerization method and different probe nucleotides were immobilized on the PEDOT film via N-hydroxysuccinimide and EDAC.HCl coupling method. The prepared grafted films were then employed for DNA detection using fluorescent, QCM, and electrochemical DNA detection methods [16]. Similarly, a study by Xu’s group reported electrochemical polymerization of poly(C_4_-EDOT-COOH) films and preparation of electrochemical chemical-/bio-sensors by covalent linkage of the polymer carboxyl group with biomolecules and layer-by-layer assembly with inorganic materials for fast and sensitive determination of catechol, ascorbic acid, Cd^2+^, acetaminophen, quercetin, epinephrine, and tryptophan via different electrochemical methods [14].

On the other hand, when ECPs were applied for manufacturing of electrochemical devices such as sensors, the properties of ECP materials, particularly at the nanometer scale, are dramatically affected by the morphology of the polymers formed [16,19,20,21,22,23]. This makes it challenging to reproduce the electrochemical readouts and their effect by the sensing targets [24,25,26]. In contrast, the intrinsic conductivities of ECPs are less sensitive toward the material morphology but more sensitive toward the charge perturbation from the environment, which can be produced by analyte or sensing targets binding. In addition, for amperometric sensory devices constructed from ECPs, the output current will increase or decrease upon analyte binding and sometimes not sufficiently large for detection of analytes due to quenching of the intrinsic conductivity of ECPs. Therefore, preparing a sensory device that will not be affected by the nano structure morphology of the ECPs used, is more sensitive to changes, and that can produce high signal output without affecting the intrinsic conductivity of ECPs is critical.

Previously, we invented a new type of conductivity based sensing system that can solve morphology related problems without affecting the intrinsic conductivity of the ECPs used [27]. For this reason, we selected poly(C_2_-EDOT-COOH) and poly(C_4_-EDOT-COOH) and studied their conductivity curve profiles at different pH. While switching the pH in the range pH 4–10, all the polymers showed horizontal conductivity shifts without affecting the intrinsic conductivity of the polymers. The shifts were independent of the electrodeposited polymers’ morphology and resulted large electrical signal output at each pH of the aqueous buffer [27]. Rather, the shifts were resulted from carboxylic acid side-chain perturbations to the main chain positive charge carriers. Between poly(C_2_-EDOT-COOH) and poly(C_4_-EDOT-COOH), the latter showed a larger horizontal shift toward more positive potential in the drain current onset potential when pH of an aqueous buffer changed from pH 4 to pH 10 [27]. These initial results inspired us to further explore and understand on the length effect of the carboxylic acid side chains toward PEDOT conductivity profile. The optimized results can offer the best materials for sensor applications with large operation window and current output. 

Herein we report the syntheses of different carboxylic acid-functionalized EDOT monomers (C_n_-EDOT-COOH) and respective polymers and the conductivity curve profiles of these electrochemical polymerized poly(C_n_-EDOT-COOH)s in acidic, neutral, and basic pH. Moreover, we also examined the conductivity shifts of poly(EDOT-OH-*co*-C_2_-EDOT-COOH)s in order to investigate how polymer composition and a small change in the negative-charge density effect the material characteristics. So, such system can be used to detect any analyte (proton, biomolecules, or other cations) that can interact with the polymer with in a particular concentration and cause a negative charge gradient surrounding the polymer backbone.

## 2. Materials and Methods 

### 2.1. Materials and Measurements

Hydroxymethyl EDOT (EDOT-OH) and Sodium hydride 60% mineral oil suspension purchased from Angene (London, England) and Sigma Aldrich (St. Louis, MO, USA), respectively. NaHCO_3_ and MgSO_4_ purchased from J. T. Baker (Avantor Performance Materials, Inc., Center Valley, PA, USA),. All other chemicals, including NaI, methyl bromoacetate, succinic anhydride, glutaric anhydride, 4-dimethylaminopyridine (DMAP), triethylamine (Et_3_N), (N-(3-dimethylaminopropyl)-N-ethylcarbodiimide hydrochloride (EDAC.HCl), dicarboxylic acids, buffer solutions, tetrabutylammonium hexafluorophosphate (*n*Bu_4_NPF_6_), and KNO_3_ were purchased from Alfa Aesar (Heysham, England). All chemicals used as received without further purification unless otherwise noted. The reactions were done using dry solvents under a nitrogen atmosphere. ^1^H and ^13^C NMR spectra were recorded with either Bruker AVA-300 or Bruker AVIII-400 spectrometers (Bruker Co., Fällanden, Switzerland) and chemical shifts were measured in δ (ppm) with residual solvent peaks as internal standards. Mass spectra were obtained on a TOF-MS spectrometer (Bruker Daltonics, Bremen, Germany). The electrochemical measurements were conducted with an Autolab PGSTAT 128N potentiostat (ECO CHEMIE BV, Utrecht, the Netherlands). The 10-µm interdigitated microelectrodes were purchased from ALS Co., Ltd (Tokyo, Japan). For electrochemical polymerization, a Ag/Ag^+^ reference electrode, a Pt wire counter electrode, and 10-µm IMEs as working electrodes were utilized. A Multimode Atomic Force Microscope (Digital Instruments, Nanoscope III, Santa Barbara, CA, USA) was utilized for polymers morphology studies.

### 2.2. Synthesis of C_n_-EDOT-COOH Monomers

#### 2.2.1. Synthesis of 4-((2,3-dihydrothieno[3,4-b][1,4]dioxin-2-yl)methoxy)-4-oxobutanoic acid (C_4_-EDOT-COOH) [27]

EDOT-OH (1.0 g, 5.81 mmol) was dissolved in CH_2_Cl_2_ (12 mL) after evacuating and backfilled the flask with nitrogen. Succinic anhydride (1.16 g, 11.6 mmol), 4-dimethylaminopyridine (DMAP; 0.065 g, 0.53 mmol) and triethylamine (Et_3_N; 1.17 mL, 0.85 g, 8.4 mmol) were dissolved in CH_2_Cl_2_ (8 mL) and added to the above solution dropwise via syringe, and stirred at room temperature overnight. The solution was washed with aqueous HCl (10%), water (until neutralized) and brine, dried (MgSO_4_), and concentrated to yield a viscous oil which solidified later to give a white solid (1.3 g, 82%). ^1^H NMR (400 MHz, CDCl_3_), δ: 6.36 (d, 1H, J = 3.6 Hz), 6.34 (d, 1H, J = 3.6 Hz), 4.40–4.31 (m, 3H), 4.23 (dd, 1H, J = 2.0, 11.6 Hz), 4.04 (dd, 1H, J = 6.8, 12.0 Hz), 2.73–2.65 (m, 4H). ^13^C NMR (100 MHz, CDCl_3_) δ: 177.9, 171.9, 141.4, 141.2, 100.3, 100.2, 71.6, 65.7, 62.8, 29.0, 28.9. 

#### 2.2.2. Typical Synthesis of C_7_- to C_16_-EDOT-COOH Monomers 

These monomers were synthesized following reported procedure [28]. EDOT-OH (1.0 g, 5.81 mmol), the respective dicarboxylic acid (2 equiv., 11.6 mmol), DMAP (0.71 g, 5.81 mmol), and N-(3-dimethylaminopropyl)-N-ethylcarbodiimide hydrochloride (EDAC∙HCl; 2.23 g, 11.6 mmol) were charged to 100 mL two-neck round-bottom flask, evacuated and backfilled with N_2_. THF and CH_2_Cl_2_ (30 mL, 1:1, v/v) were added and stirred for 4 h at room temperature. The majority of the solvent was removed under rotary evaporator; the residue was dissolved in CH_2_Cl_2_ and washed with NaHCO_3_, brine and DI water. The aqueous phase was extracted with CH_2_Cl_2_ (3×); the organic phase was dried (MgSO_4_) and concentrated. The crude product was purified by a silica gel column using hexane/ethyl acetate (7:3, v/v) eluent. 

#### 2.2.3. 7-((2,3-dihydrothieno[3,4-b][1,4]dioxin-2-yl)methoxy)-7-oxoheptanoic acid (C_7_-EDOT-COOH) 

The product was purified as viscous oil, which later solidified when kept in a refrigerator to yield a white solid (1.40 g, 77%). ^1^H NMR (400 MHz, CDCl_3_), δ: 6.36 (d, 1H, J = 4.0 Hz), 6.34 (d, 1H, J = 3.6 Hz), 4.40–4.34 (m, 1H), 4.31 (d, 2H, J = 4.8 Hz), 4.23 (dd, 1H, J = 2.0, 11.6 Hz), 4.04 (dd, 1H, J = 6.8, 11.6 Hz), 2.38 (q, 4H, J = 7.2 Hz), 1.70–1.62 (m, 4H), 1.43–1.39 (m, 2H). ^13^C NMR (100 MHz, CDCl_3_) δ: 179.6, 173.3, 141.4, 141.2, 100.3, 100.2, 71.7, 65.8, 62.4, 33.92, 33.9, 28.6, 24.6, 24.4. HR (TOF MS AP^+^) m/z: [M+H]^+^ Calcd for C_14_H_19_O_6_S 315.0902; Found 315.0911.

#### 2.2.4. 9-((2,3-dihydrothieno[3,4-b][1,4]dioxin-2-yl)methoxy)-9-oxononanoic acid (C_9_-EDOT-COOH) 

The product was purified as viscous oil, which latter solidified when kept in a refrigerator to yield a white solid (0.90 g, 45%). ^1^H NMR (300 MHz, CDCl_3_), δ: 6.37 (d, 1H, J = 4.8 Hz), 6.36 (d, 1H, J = 4.8 Hz), 4.42–4.36 (m, 1H), 4.33 (d, 2H, J = 6.4 Hz), 4.26 (dd, 1H, J = 3.2, 15.6 Hz), 4.06 (dd, 1H, J = 9.6, 15.6 Hz), 2.37 (td, 4H, J = 5.2, 10.0 Hz), 1.66–1.64 (b, 4H), 1.34 (b, 6H). ^13^C NMR (75 MHz, CDCl_3_) δ: 179.9, 173.5, 141.4, 141.3, 100.3, 100.1, 71.7, 65.8, 62.3, 34.1 (2C), 29.0 (3C), 24.9, 24.7. HR (TOF MS ES^+^) m/z: [M+Na]^+^ Calcd for C_16_H_22_O_6_SNa 365.1035; Found 365.1043.

#### 2.2.5. 14-((2,3-dihydrothieno[3,4-b][1,4]dioxin-2-yl)methoxy)-14-oxotetradecanoic acid (C_14_-EDOT-COOH) 

The product was purified as white powder (1.14 g, 48%). ^1^H NMR (400 MHz, CDCl_3_), δ: 6.33 (d, 1H, J = 3.6 Hz), 6.31 (d, 1H, J = 3.6 Hz), 4.37–4.28 (m, 1H), 4.27 (d, 2H, J = 2.8 Hz), 4.21 (dd, 1H, J = 1.6, 11.6 Hz), 4.01 (dd, 1H, J = 7.2, 12.0 Hz), 2.33 (td, 4H, J = 4.4, 7.6 Hz), 1.64–1.57 (m, 4H), 1.26–1.24 (b, 16H). ^13^C NMR (100 MHz, CDCl_3_) δ: 179.9, 173.6, 141.4, 141.3, 100.2, 100.1, 71.7, 65.9, 62.3, 34.2 (2C), 29.7 (2C), 29.6 (2C), 29.4 (2C), 29.3, 29.2, 25.1, 24.9. HR (TOF MS ES^+^) m/z: [M+Na]^+^ Calcd for C_16_H_22_O_6_SNa 365.1035; Found 365.1043. HR (TOF MS ES^+^) m/z: [M+Na]^+^ Calcd for C_21_H_32_O_6_SNa 435.1817; Found 435.1815.

#### 2.2.6. 16-((2,3-dihydrothieno[3,4-b][1,4]dioxin-2-yl)methoxy)-16-oxohexadecanoic acid (C_16_-EDOT-COOH) 

The product was purified as white powder (1.06 g, 42%). ^1^H NMR (300 MHz, CDCl_3_), δ: 6.38 (d, 1H, J = 4.0 Hz), 6.36 (d, 1H, J = 4.0 Hz), 4.42–4.36 (m, 1H), 4.33 (dd, 2H, J = 2.4, 5.2 Hz), 4.26 (dd, 1H, J = 2.0, 12.0 Hz), 4.06 (dd, 1H, J = 7.2, 11.6 Hz), 2.37 (td, 4H, J = 3.6, 7.6 Hz), 1.69 – 1.61 (m, 4H), 1.27 (b, 20H). ^13^C NMR (75 MHz, CDCl_3_) δ: 179.7, 173.7, 141.4, 141.3, 100.3, 100.1, 71.7, 65.9, 62.3, 34.3, 34.2, 29.8 (4C), 29.6 (2C), 29.4, (2C), 29.3, 29.2, 25.1, 24.9. HR (TOF MS ES^+^) m/z: [M+Na]^+^ Calcd for C_23_H_36_O_6_SNa 463.2130; Found 463.2125.

### 2.3. Electrochemical Polymerization of C_n_-EDOT-COOHs

The interdigitated microelectrodes (IMEs) were cleaned with a piranha solution (75% conc. H_2_SO_4_ and 25% H_2_O_2_ (30% aq. solution), v/v) before utilizing for electrochemical polymerization. To do so, the IMEs were immersed in hot, newly prepared piranha solution for 5 seconds and immediately quenched with DI water for 2 minutes. Further washed with DI water for 1 minute, dried with air blow and used for polymerization. During electrochemical polymerization, a Ag/Ag^+^ reference electrode and a Pt wire counter electrode were utilized. Each electrolyte solution was degassed before performing the electrochemical polymerizations. The electrochemical polymerizations on 10 µm IMEs were performed using 10 mM of each monomer solution in anhydrous CH_3_CN and 0.1 M *n*Bu_4_NPF_6_ as supporting electrolyte. The polymers were electrodeposited on IMEs by potential sweeps between −0.8 and 1.25 V over 2 cycles at room temperature, with a scan rate of 100 mV/s. The copolymers were also electrodeposited by similar method with the exception of changes in monomer composition. The electrodeposited polymer films were washed with CH_3_CN and air-dried before used for drain current measurements.

### 2.4. Drain Current Measurement

The conductivity of all homopolymers and copolymers electrodeposited on IMEs were measured in a buffer solution containing 0.1 M of KNO_3_ supporting electrolyte at a scan rate of 5 mV/s with an offset of 100 mV between two working electrodes following reported procedure [27]. The conductivity was measured by a four-point probe method in which the polymers deposited on the IMEs are connected with two adjacent sets of electrodes (source and drain electrodes). A fixed potential difference (100 mV) was set between the two electrodes to create a potential gradient across the polymer film. The oxidation state of the polymer changes when a potential is swept relative to reference electrode. At some potential, the polymer becomes conductive and current flows between the two sets of electrodes and this current is measured which is termed as drain current. This drain current is proportional to the conductivity of the polymer. Hence, the conductivity of each poly(EDOT-COOH) polymer was recorded and analyzed.

## 3. Results and Discussion

### 3.1. Synthesis of EDOT-COOH Monomers and Polymers with Different Side-Chain Length

**Scheme 1:**
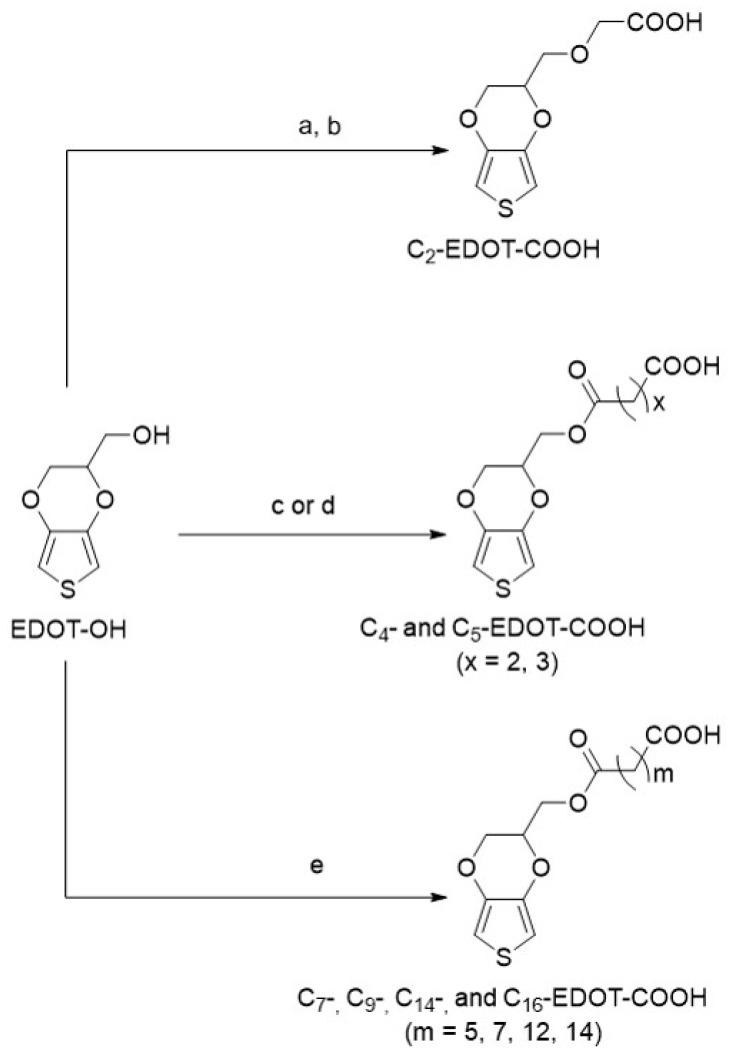
Synthesis routes to C_n_-EDOT-COOH monomers: (a) NaI, NaH, CH_2_BrCOOCH_3_, THF; (b) aq. NaOH (2 M), THF; (c and d) succinic anhydride or glutaric anhydride, DMAP, Et_3_N, CH_2_Cl_2_ (e) dicarboxylic acid, EDAC, DMAP, THF/CH_2_Cl_2_.

We initiated the synthesis of carboxylic acid-functionalized EDOTs (EDOT-COOH) from known hydroxymethyl-functionalized EDOT (EDOT-OH) [29] as shown in Scheme 1. In general, EDOT-COOHs can be synthesized from EDOT-OH through etherification of the hydroxyl group by nucleophilic substitution using an alkylbromide containing protected carboxylic acids and further treatment with aq. base, or through esterification method. In our study, C_2_-EDOT-COOH was synthesized by reacting EDOT-OH with methylbromoacetate through substitution reaction to give EDOT-COOCH_3_ and further treatment with aq. NaOH resulted the product [27]. C_4_- and C_5_-EDOT-COOHs were synthesized from EDOT-OH, and succinic anhydride and glutaric anhydride, respectively, following the literature procedure [27]. Instead of using anhydrides, C_7_-, C_9_-, C_14_-, and C_16_-EDOT-COOH were synthesized from EDOT-OH and the respective cheaper dicarboxylic acids through esterification method, following reported procedure by our group [28]. This method afforded moderate to higher yields of carboxyl acid-functionalized EDOTs with single step. The monomers were then characterized by NMR and mass spectroscopy (Figures S4–S17).

Electrochemical polymerization of each carboxylic acid-functionalized EDOT monomer on microelectrodes (with 10 µm width of arrays and 5 µm interval between each array) yielded blue polymer films. It was performed by applying cyclic potential from −0.8 to 1.25 V in CH_3_CN solution containing 10 mM of the respective monomer and 0.1 M *n*Bu_4_PF_6_ as supporting electrolyte for 2 cycles. The CH_3_CN-rinsed and air-dried poly(C_n_-EDOT-COOH) polymer films were directly utilized for in situ conductivity studies in aqueous buffer solution using KNO_3_ as supporting electrolyte. All polymers showed intrinsic electrical conductivity at the same order of magnitude as that of poly(EDOT-OH) (~100 S/cm) once the polymers were electrochemically doped. 

**Figure 1:**
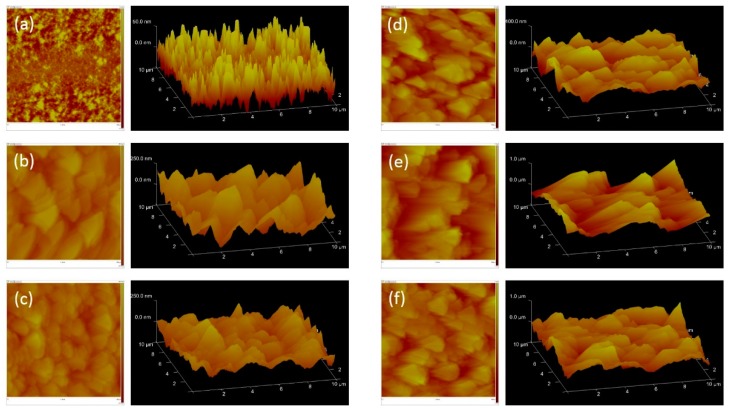
AFM topographic (left) and 3D (right) images of (**a**) poly(C_4_-EDOT-COOH); (**b**) poly(C_5_-EDOT-COOH); (**c**) poly(C_7_-EDOT-COOH); (**d**) poly(C_9_-EDOT-COOH); (**e**) poly(C_14_-EDOT-COOH); (**f**) poly(C_16_-EDOT-COOH). The scan size is 10 μm × 10 μm.

The polymer formation on the IMEs was confirmed from the polymerization cyclic voltammetry of each polymer with a sharp rise in the measured current for potential higher than ~1.1 V (vs. Ag/Ag^+^ reference electrode) (Figure S1). In addition, the surface morphologies of the polymers were investigated by atomic force microscope (AFM) (Figure 1). AFM images indicated an increase in the roughness of the polymer films going from poly(C_4_-EDOT-COOH) to poly(C_16_-EDOT-COOH) with respect to the carboxyl acid-side-chain length. All the polymers showed rough surfaces with poly(C_4_-EDOT-COOH) relatively denser.

### 3.2. Effect of Carboxylic Acid Side-Chain Length on the Poly(EDOT-COOH) Conductivity

**Figure 2:**
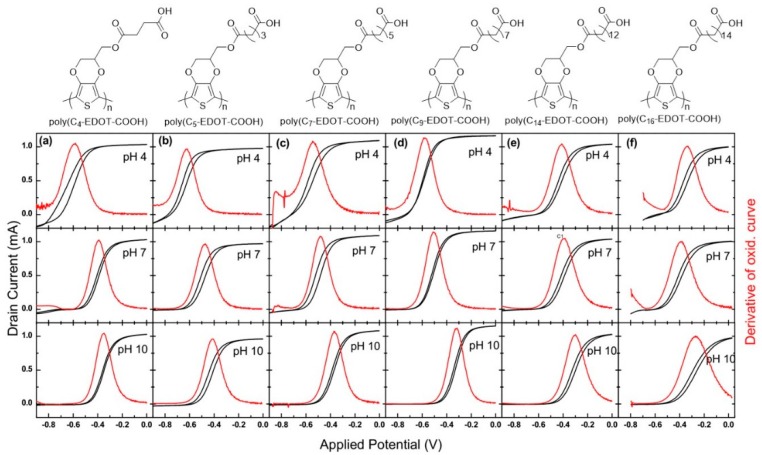
Drain current measurement of (**a**) poly(C_4_-EDOT-COOH); (**b**) poly(C_5_-EDOT-COOH); (**c**) poly(C_7_-EDOT-COOH); (**d**) poly(C_9_-EDOT-COOH); (**e**) poly(C_14_-EDOT-COOH); and (**f**) poly(C_16_-EDOT-COOH) electrodeposited on 10 µm IMEs in 10 mM of pH 4, pH 7 and pH 10 buffer solutions using 0.1 M of KNO_3_ as supporting electrolyte. The red line indicates the first derivative of the oxidation drain current sweep (*E*_onset_).

When we applied a 100 mV offset between the two sets of interdigitated working electrodes, we could monitor the drain current that passed through the poly(C_n_-EDOT-COOH) (n = 4, 5, 7, 9, 14, and 16) covered electrode at different applied potential as shown in Figure 2 (see Figures S1 and S2 for electrochemical polymerization CV and individual conductivity curves). In order to keep the ionic strength constant, we added 0.1 M KNO_3_ to the buffer solution at different pH. A typical drain current curve started to increase at certain potential and reached plateau rapidly, which represents the doping of PEDOTs by electrochemical oxidation.

**Figure 3:**
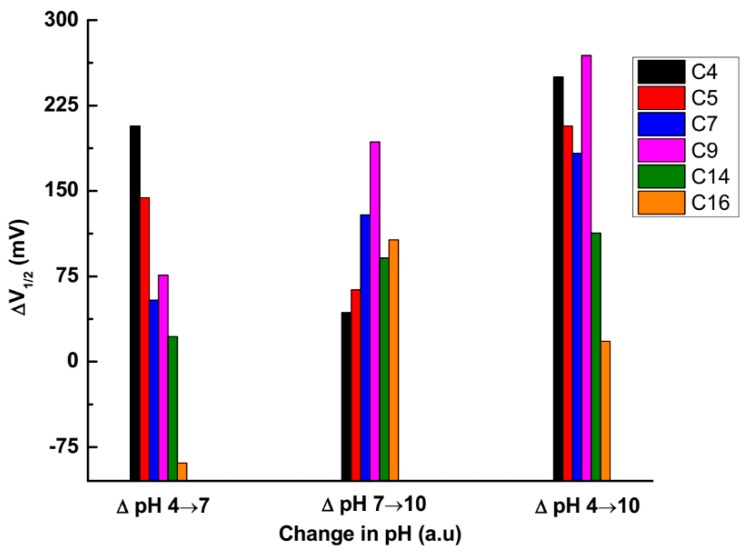
Comparison of change in *E*_onset_ (ΔV) values for conductivity curve of poly(C_n_-EDOT-COOH)s while changing pH of an aqueous buffer from pH 4 to pH 7, pH 7 to pH 10, and pH 4 to pH 10 (the ΔV values are shown in Table 1).

The conductivity curve profiles of poly(C_n_-EDOT-COOH)s in an aqueous buffer of pH 4, pH 7, and pH 10 were summarized in Table 1 and Figure 3. The onset potential, *E*_onset_, was defined as the average of the maximum oxidation and reduction peak potentials obtained from the first derivative of the drain current curve. All polymers showed a horizontal shift in the *E*_onset_ of the drain current curve towards more positive potential when the pH of an aqueous buffer changed from pH 4 to pH 7 and pH 7 to pH 10. Poly(C_4_-EDOT-COOH) and poly(C_5_-EDOT-COOH) showed larger shifts in *E*_onset_ values as 207 and 144 mV, respectively, when the pH of an aqueous buffer changed from pH 4 to pH 7. In contrast, the largest shifts in the drain current curves when the pH of buffer changed from pH 7 to pH 10 occurred in poly(C_7_-EDOT-COOH)s and poly(C_9_-EDOT-COOH)s, resulting in ΔV of 129 and 193 mV, respectively. When we compared the conductivity curve at pH 4 and pH 10, we observed large differences in poly(C_4_-, C_5_-, C_7_-and C_9_-EDOT-COOH)s and almost no change in poly(C_16_-EDOT-COOH).

### 3.3. The Factors that Affect the Conductivity Shift 

**Scheme 2:**
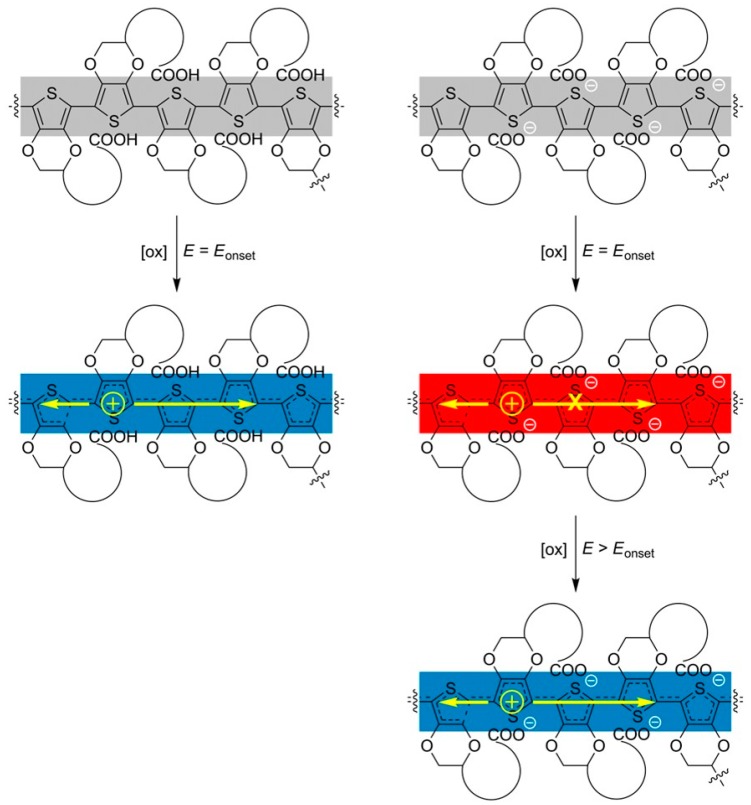
Representation illustrated the side-chain deprotonation that perturbed the intrinsic conductivity of PEDOTs.

There is more than one factor to explain why the conductivity shifts and how chain length effects the conductivity curve. We have previously explained how side-chain deprotonation effects the conductivity curve. It is different from those of polyanilines, polypyrroles, and polythiophenes, where the maximum conductivity may decrease as a result of reduced resonance stabilization during deprotonation of the polymer backbone [30,31,32,33]. As illustrated in Scheme 2, deprotonation of the carboxylic acid group in poly(C_n_-EDOT-COOH) polymers occurs in concomitant with the generation of positive charge carriers during electrochemical oxidation of the polymer backbone. Hence, charge neutrality maintained and similar intrinsic conductivity could be achieved by applying a more positive potential that increases the mobile charge carrier density, as observed for a shift in the *E*_onset_ of drain current curve without decreasing maximum conductivity.

Another factor on the differences of *E*_onset_ is the acid dissociation property (p*K*_a_) at the solid–liquid interface of carboxylic acids with different side chain lengths. In general, ionization of a carboxylic acid to release a proton decreases as the length of the alkyl chain increases [34]. This difference in the ionization property of the carboxylic acids at different pH resulted in different negative charge density around the polymer backbone. Hence, the potential required to overcome charge neutrality in the doped state and bring the polymer to its conductive state differs. This assumption was clearly supported by comparing the values of ΔV. The values of ΔV (207, 144, 54, and 22 mV for poly(C_4_-, C_5_-, C_7_-, and C_14_-EDOT-COOH), respectively) decreased with increasing alkyl chain length when pH changed from 4 to 7. On the other hand, values of ΔV (43, 63, 129, and 193 mV for poly(C_4_-, C_5_-, C_7_-, and C_9_-EDOT-COOH), respectively) increased with increasing alkyl chain length when pH changed from 7 to 10. A typical carboxylic acid group becomes deprotonated in the buffer of pH 5. This was why poly(C_4_-EDOT-COOH) and poly(C_5_-EDOT-COOH) showed large conductivity shift when buffer pH changed from 4 to 7. However, surface-bound long-chain carboxylic acid could increase the pKa to 6.5–9 [34]. Therefore, large conductivity shift occurred when buffer pH changed from 7 to 10 in the case of poly(C_7_-EDOT-COOH)s, poly(C_9_-EDOT-COOH) and poly(C_14_-EDOT-COOH).

In addition, the length of the carboxylic acid side-chains could also affect the conductivity profile through two other mechanisms. Long side-chain would allow the negative-charge bending back to be closer to the main polymer backbone, enhancing the self-doping effect [35,36]. In our experiments, poly(C_9_-EDOT-COOH) showed the largest conductivity shift (269 mV) between pH 4 and pH 10. This could be explained by poly(C_9_-EDOT-COOH) exhibited the optimized side-chain length for the bending back effect. As a result, more positive potential required to bring this polymer into its conductive state at higher pH.

The other mechanism that changes the conductivity profile is the effective conjugation length of the PEDOTs. The *E*_onset_ values of poly(C_14_-EDOT-COOH) and poly(C_16_-EDOT-COOH) in pH 4 buffer were more positive compared to other poly(EDOT-COOH)s. This could be attributed to nonplanar conformations induced by bulky side chains, resulting in lower conjugation length of the polymers. Therefore, higher potentials were required to oxidize the polymers [37,38]. Only small shifts in the conductivity curve were observed for poly(C_14_-EDOT-COOH) (113 mV) and poly(C_16_-EDOT-COOH) (18 mV) between pH 4 and pH 10.

### 3.4. Compositional Effect on the Copolymer Conductivity

**Figure 4:**
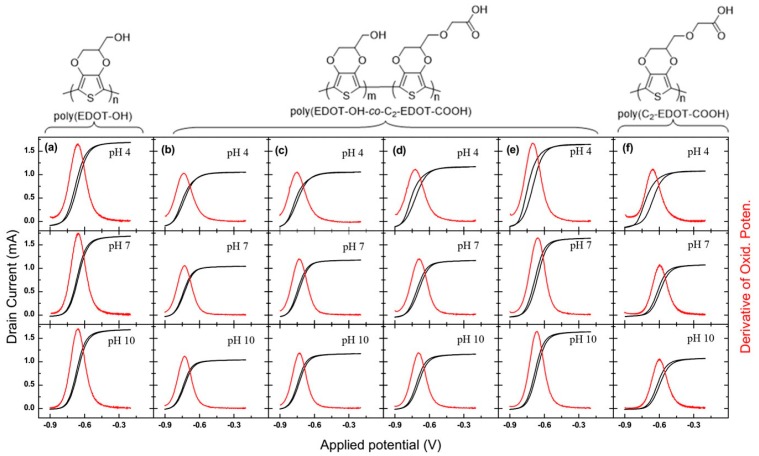
Drain current measurement of (**a**) poly(EDOT-OH) (**b**–**e**) poly(EDOT-OH-*co*-C_2_-EDOT-COOH) with monomers ratio (EDOT-OH/ C_2_-EDOT-COOH) of 80/20, 60/40, 40/60, and 20/80, respectively, (**f**) poly(C_2_-EDOT-COOH). The red lines indicate the first derivative of the oxidation drain current sweep (*E*_onset_).

The property of a material can be modulated by mixing different monomers to form a copolymer to accomplish desired properties [39]. As we demonstrated [27], poly(C_2_-EDOT-COOH) showed a small shift on *E*_onset_ with changing the aqueous buffer from pH 4 to pH 10 while poly(EDOT-OH) showed almost no shifts. It would be intriguing to know how the side-chain density of negative-charged carboxylate would affect the conductivity profile of PEDOTs. Copolymers poly(EDOT-OH-*co*-C_2_-EDOT-COOH)s were deposited onto interdigitated microelectrodes in the same way as homopolymers through electrochemical polymerization in the presence of EDOT-OH/C_2_-EDOT-COOH monomer mixture at a molar ratio of 100/0, 80/20, 60/40, 40/60, 20/80, and 0/100 (Figure S3). Poly(EDOT-OH-*co*-C_2_-EDOT-COOH)s, also showed similar conductivity curves as homopolymers (Figure 4). 

**Figure 5:**
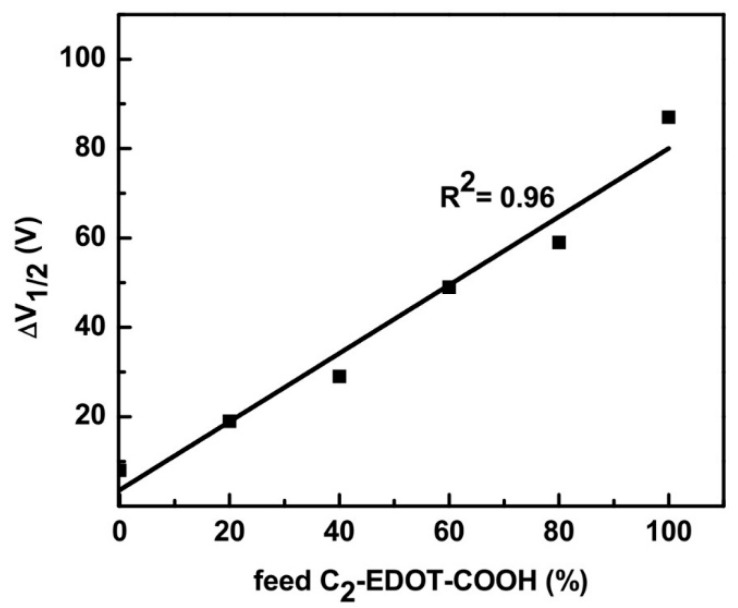
Differences of onset potential in buffer solution at pH 4 and pH 10 from poly(EDOT-OH-*co*-C_2_-EDOT-COOH) with different feed C_2_-EDOT-COOH composition.

As long as the copolymer includes carboxylic acid functional groups, we observed conductivity shifts toward more positive potential when the buffer solution switched from pH 4 to pH 10. As the feed composition of C_2_-EDOT-COOH increased from 0 to 100%, larger shifts were displayed (8 mv for 0%, 19 mV for 20%, 29 mV for 40%, 49 mV for 60 %, 59 mV for 80%, 87 mV for 100%) and a linear relationship between feed composition and *E*_onset_ shifts between pH 4 and pH 10 as shown in Figure 5. The results confirmed that even small charge perturbation at the side-chains can be sensitively detected by the main chain conductivity profile. This could lead to highly sensitive sensor design through the side-chain charge perturbation.

## 4. Conclusions

In conclusion, we observe that the deprotonation-induced conductivity shifts of poly(C_n_-EDOT-COOH)s are influenced by the side-chain lengths. This observation can be attributed to three different factors: (1) the acid dissociation property (p*K*_a_) at the solid–liquid interface of carboxylic acids, (2) self-doping effect, and (3) effective conjugation length of the PEDOTs. As a result, largest horizontal conductivity shift of 269 mV is observed from poly(C_9_-EDOT-COOH) when the pH of an aqueous buffer switches from 4 to 10. In the case of poly(EDOT-OH-*co*-C_2_-EDOT-COOH) copolymer, we observe a linear relationship between feed composition of monomers and onset potential shifts from pH 4 to pH 10. It confirms that even small side-chain charge perturbation can be detected by main-chain conductivity profile. Our study suggested the optimized material to be integrated for developing future conductivity-based chemical or biological sensors from charge modulation, minimizing morphology effects.

## Figures and Tables

**Table 1:** *E*_onset_ and ΔV of poly(C_n_-EDOT-COOH)s at different pH.

Poly(C_n_-EDOT-COOH)	*E*_onset_ (V) ^a^			ΔV (V)		
	pH 4	pH 7	pH 10	ΔV_4→7_	ΔV_7→10_	ΔV_4→10_
poly(C_4_-EDOT-COOH)	−0.606	−0.399	−0.356	0.207	0.043	0.250
poly(C_5_-EDOT-COOH)	−0.635	−0.491	−0.428	0.144	0.063	0.207
poly(C_7_-EDOT-COOH)	−0.557	−0.503	−0.374	0.054	0.129	0.183
poly(C_9_-EDOT-COOH)	−0.594	−0.518	−0.325	0.076	0.193	0.269
poly(C_14_-EDOT-COOH)	−0.425	−0.403	−0.312	0.022	0.091	0.113
poly(C_16_-EDOT-COOH)	−0.331	−0.420	−0.313	−0.089	0.107	0.018

^a^*E*_onset_, is defined as the average of the maximum oxidation and reduction peak potentials obtained from the first derivative of the drain current curve.

## Supplementary Materials

The following are available online at https://www.mdpi.com/2073-4360/11/4/659/s1, Figure S1. Electrochemical polymerization of C_4_-, C_5_-, C_7_-, C_9_-, C_14_- and C_16_-EDOT-COOHs on 10 µm IMEs from 10 mM of each monomer and 0.1 M nBu_4_NPF_6_ supporting electrolyte dissolved in anhydrous CH_3_CN by potential sweeps between -0.8 and 1.25 V. Ag/Ag+ reference electrode and Pt wire counter electrodes utilized for the polymerization; Figure S2. Drain current curve (left) and *E*_onset_ values (right) of (A) poly(C_4_-EDOT-COOH); (B) poly(C_5_-EDOT-COOH); (C) poly(C_7_-EDOT-COOH); (D) poly(C_9_-EDOT-COOH); (E) poly(C_14_-EDOT-COOH); and F) poly(C_16_-EDOT-COOH) electrodeposited on 10-µm IMEs in a buffer solution of pH 4, pH 7 and pH 10 using 0.1 M KNO_3_ as supporting electrolyte; Figure S3. Electrochemical polymerization (left) and drain current measurement (right) of poly(EDOT-OH-*co*-C_2_-EDOT-COOH) in monomer ratio of (A) 100/0; (B) 80/20; (C) 60/40; (D) 40/60, (E) 20/80; and (F) 0/100%. The dotted lines (right) are the 1^st^ derivative of the reduction curve at pH 4 and pH 10; Figure S4-S13. ^1^H and ^13^C NMR spectra of C_n_-EDOT-COOH monomers; Figure S14–S17. Mass Spectra of C_n_-EDOT-COOH monomers.
