# Supplementary material for: Deprotonation-Induced Conductivity Shift of Polyethylenedioxythiophenes in Aqueous Solutions: The Effects of Side-Chain Length and Polymer Composition
Source: Polymers (Basel). 2019 Apr 10;11(4):659. doi: 10.3390/polym11040659 (PMC6523877; doi:10.3390/polym11040659)
Supplement: Supplementary file 1 [file polymers-11-00659-s001.pdf]

## Supplementary Information

# Deprotonation Induced Conductivity Shift of Polyethylenedioxythiophenes in Aqueous Solutions: the Effects of Side-Chain Length and Polymer Composition

Hailemichael Ayalew [1,2,3], Tian-lin Wang [1] and Hsiao-hua Yu [1,2,*]

[1] Smart Organic Materials Laboratory, Institute of Chemistry, Academia Sinica, 128 Academic Road, Sec. 2, Nankang, Taipei 115, Taiwan.

[2] Taiwan International Graduate Program (TIGP), Sustainable Chemical Science and Technology (SCST), Academia Sinica, Taipei 115, Taiwan.

[3] Department of Applied Chemistry, National Chiao Tung University, Hsinchu 300, Taiwan.

* Correspondence: bruceyu@gate.sinica.edu.tw.

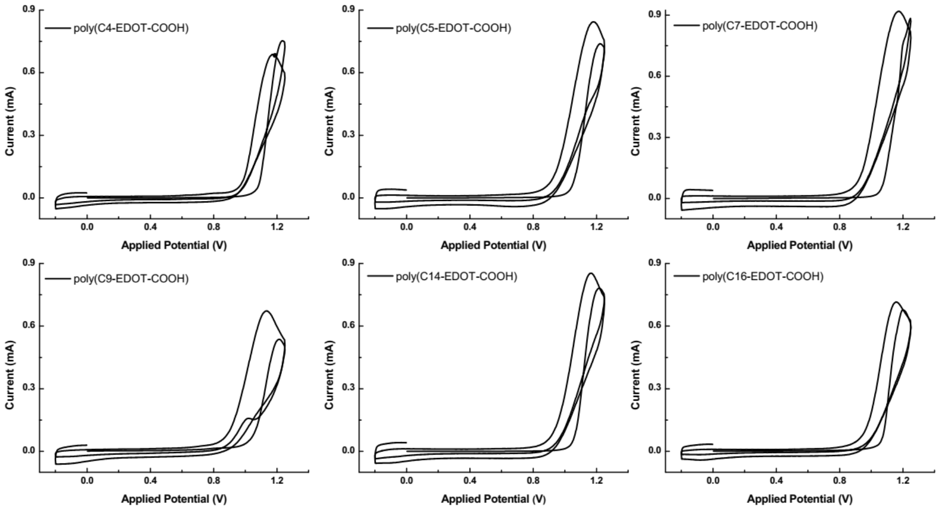


**Figure S1.** Electrochemical polymerization of $C_4$-, $C_5$-, $C_7$-, $C_9$-, $C_{14}$- and $C_{16}$-EDOT-COOHs on 10-μm IMEs from 10 mM of each monomer and 0.1 M $nBu_4NPF_6$ supporting electrolyte dissolved in anhydrous $CH_3CN$ by potential sweeps between -0.8 and 1.25 V. $Ag/Ag^+$ reference electrode and Pt wire counter electrodes utilized for the polymerization.

A. Poly($C_4$-EDOT-COOH)

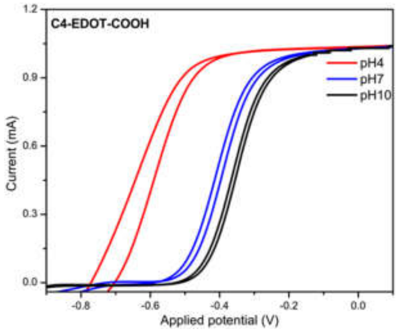


| pH | 4 | 7 | 10 | $\Delta V_{1/2\ 4\rightarrow10}$ |
|---|---|---|---|---|
| $V_{ox}$ | -0.582 | -0.390 | -0.353 | 0.229 |
| $V_{red}$ | -0.629 | -0.407 | -0.358 | 0.271 |
| $V_{1/2}$ | -0.606 | -0.399 | -0.356 | 0.250 |

B. Poly($C_5$-EDOT-COOH)

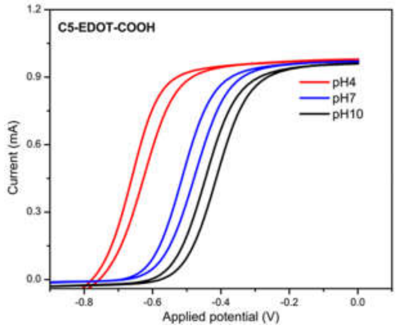


| pH | 4 | 7 | 10 | $\Delta V_{1/2\ 4\rightarrow10}$ |
|---|---|---|---|---|
| $V_{ox}$ | -0.618 | -0.471 | -0.413 | 0.205 |
| $V_{red}$ | -0.652 | -0.510 | -0.442 | 0.210 |
| $V_{1/2}$ | -0.635 | -0.491 | -0.428 | 0.207 |

C. Poly($C_7$-EDOT-COOH)

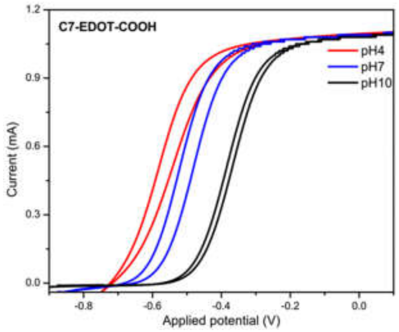


| pH | 4 | 7 | 10 | $\Delta V_{1/2\ 4\rightarrow10}$ |
|---|---|---|---|---|
| $V_{ox}$ | -0.540 | -0.484 | -0.367 | 0.173 |
| $V_{red}$ | -0.574 | -0.521 | -0.381 | 0.193 |
| $V_{1/2}$ | -0.557 | -0.503 | -0.374 | 0.183 |

D. Poly($C_9$-EDOT-COOH)

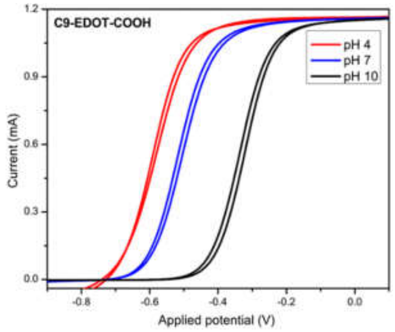


| pH | 4 | 7 | 10 | $\Delta V_{1/2\ 4\rightarrow10}$ |
|---|---|---|---|---|
| $V_{ox}$ | -0.590 | -0.511 | -0.315 | 0.275 |
| $V_{red}$ | -0.597 | -0.524 | -0.335 | 0.262 |
| $V_{1/2}$ | -0.594 | -0.518 | -0.325 | 0.269 |

E. Poly($C_{14}$-EDOT-COOH)

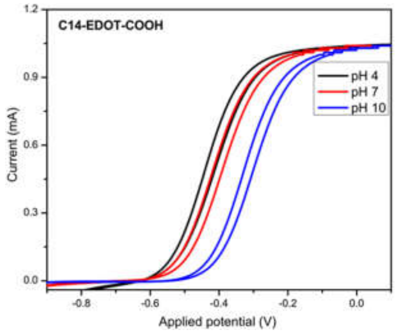


| pH | 4 | 7 | 10 | $\Delta V_{1/2\ 4\rightarrow10}$ |
|---|---|---|---|---|
| $V_{ox}$ | -0.412 | -0.392 | -0.299 | 0.113 |
| $V_{red}$ | -0.438 | -0.414 | -0.324 | 0.114 |
| $V_{1/2}$ | -0.425 | -0.403 | -0.312 | 0.113 |

F. Poly($C_{16}$-EDOT-COOH)

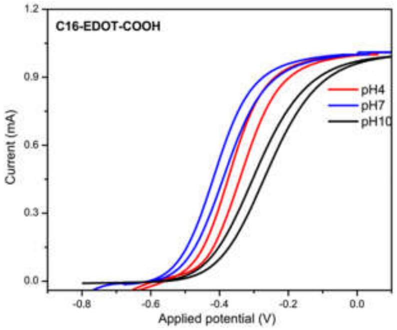


| pH | 4 | 7 | 10 | $\Delta V_{1/2\ 4\rightarrow10}$ |
|---|---|---|---|---|
| $V_{ox}$ | -0.278 | -0.402 | -0.304 | -0.017 |
| $V_{red}$ | -0.375 | -0.438 | -0.321 | 0.054 |
| $V_{1/2}$ | -0.331 | -0.420 | -0.313 | 0.018 |

**Figure S2**. Drain current curve (left) and $E_{onset}$ values (right) of A) poly($C_4$-EDOT-COOH); B) poly($C_5$-EDOT-COOH); C) poly($C_7$-EDOT-COOH); D) poly($C_9$-EDOT-COOH); E) poly($C_{14}$-EDOT-COOH); and F) poly($C_{16}$-EDOT-COOH) electrodeposited on 10-µm IMEs in a buffer solution of pH 4, pH 7 and pH 10 using 0.1 M $KNO_3$ as supporting electrolyte.

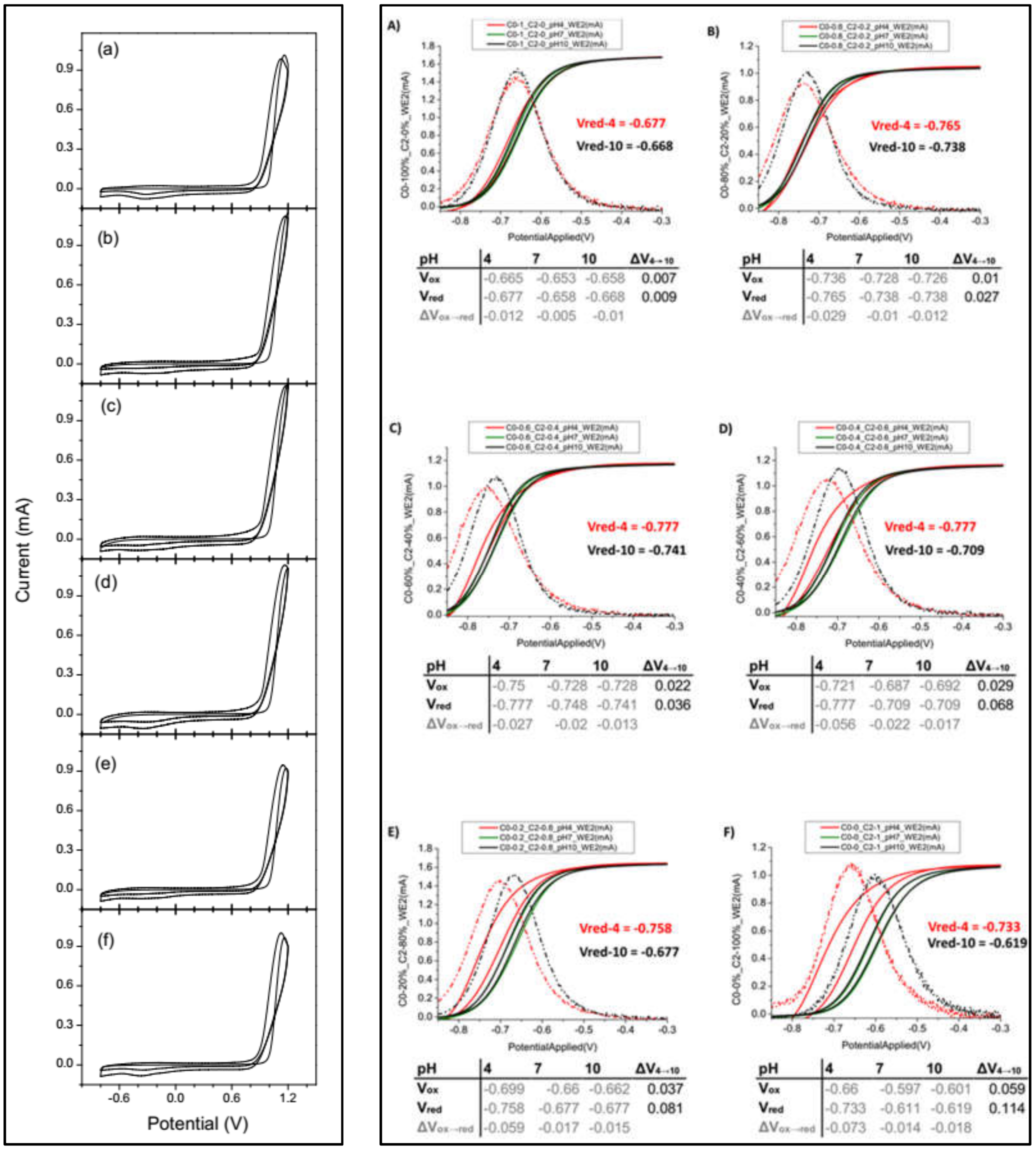


**Figure S3**. Electrochemical polymerization (left) and drain current measurement (right) of poly(EDOT-OH-*co*-$C_2$-EDOT-COOH) in monomer ratio of A) 100,0; B) 80,20; C) 60,40; D) 40,60 E) 20,80; F) 0,100%. The dotted lines (right) are the 1$^{st}$ derivative of the reduction curve at pH 4 and pH 10.

### $^{1}H$ and $^{13}C$ NMR spectra of monomers

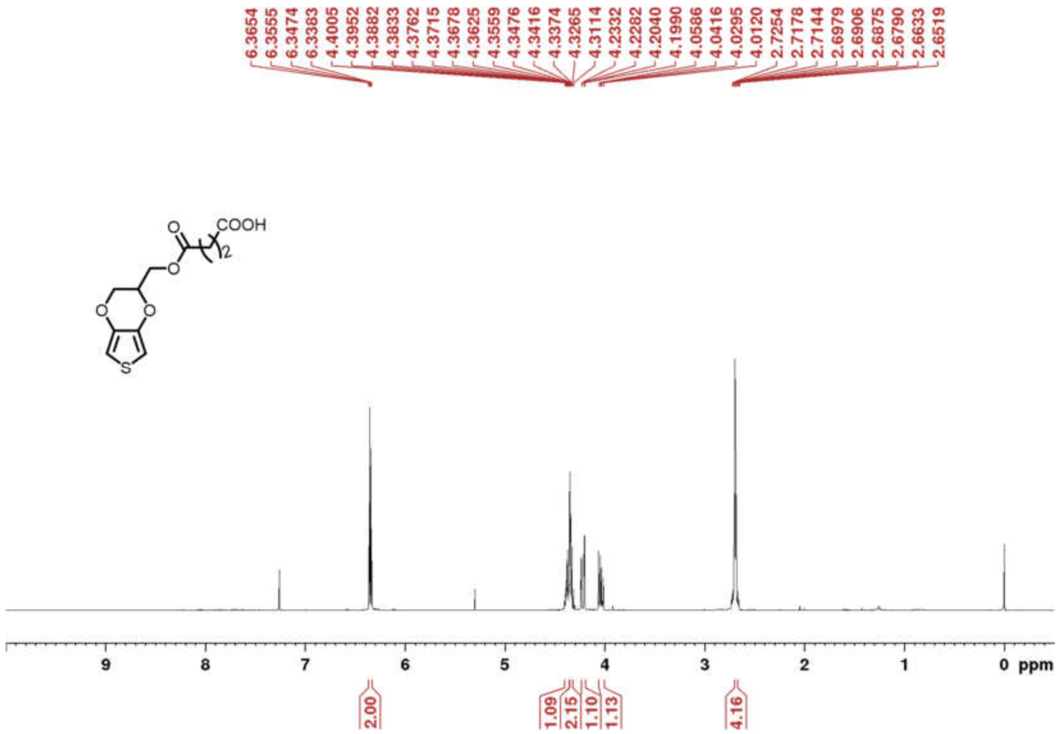

**Figure S4.** $^{1}H$ NMR spectrum of $C_4$-EDOT-COOH (400 MHz, $CDCl_3$)

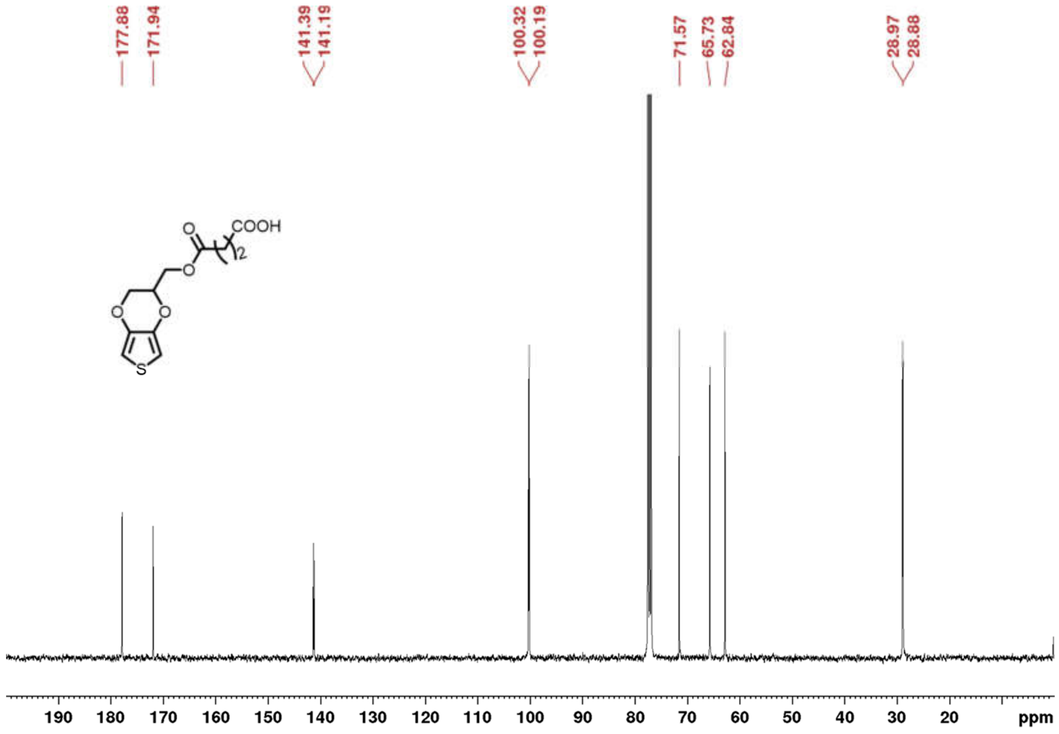

**Figure S5.** $^{13}C$ NMR spectrum of $C_4$-EDOT-COOH (100 MHz, $CDCl_3$)

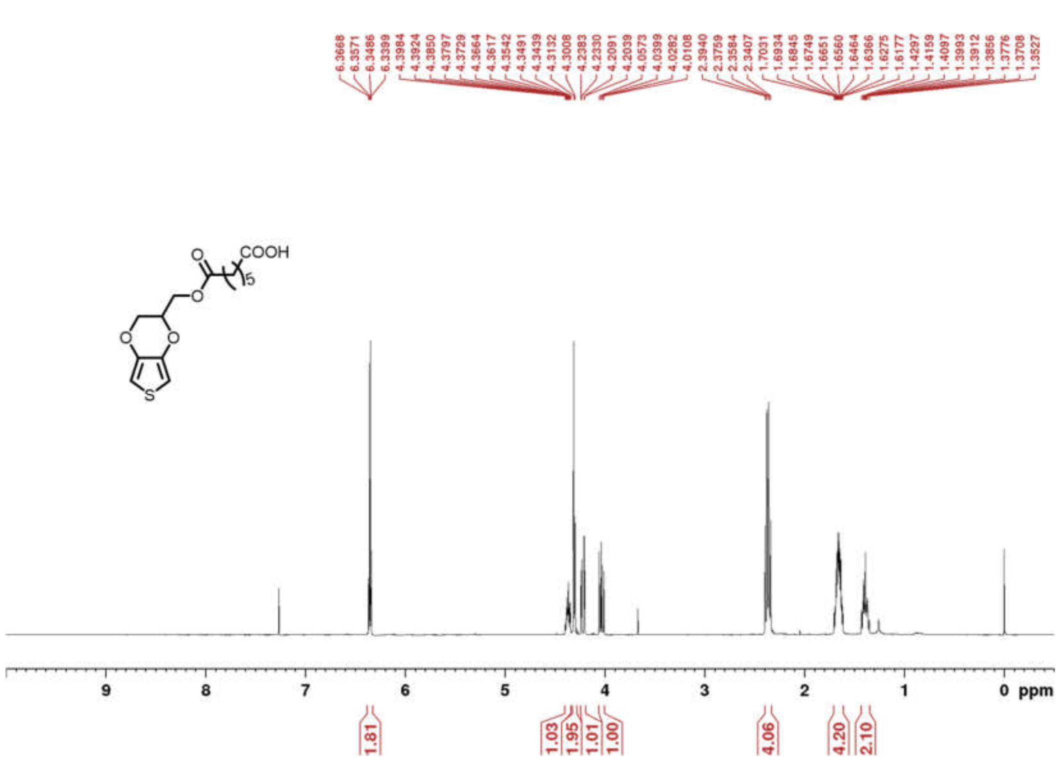

**Figure S6.** $^{1}$H NMR spectrum of $C_7$-EDOT-COOH (400 MHz, $CDCl_3$)

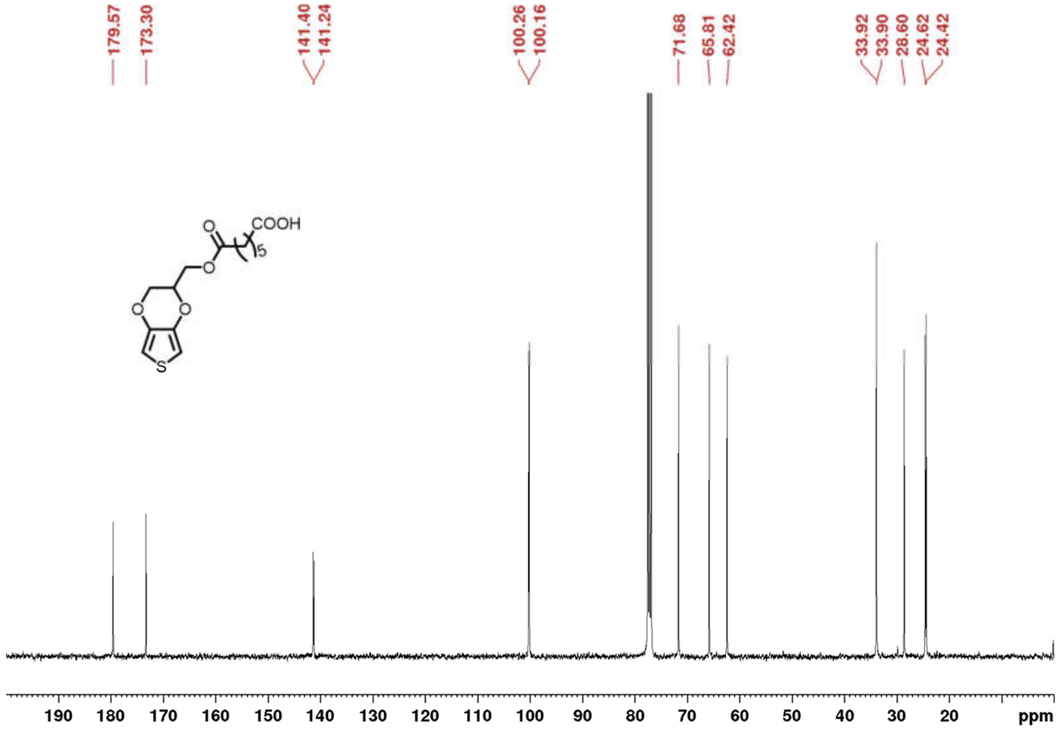

**Figure S7.** $^{13}$C NMR spectrum of $C_7$-EDOT-COOH (100 MHz, $CDCl_3$)

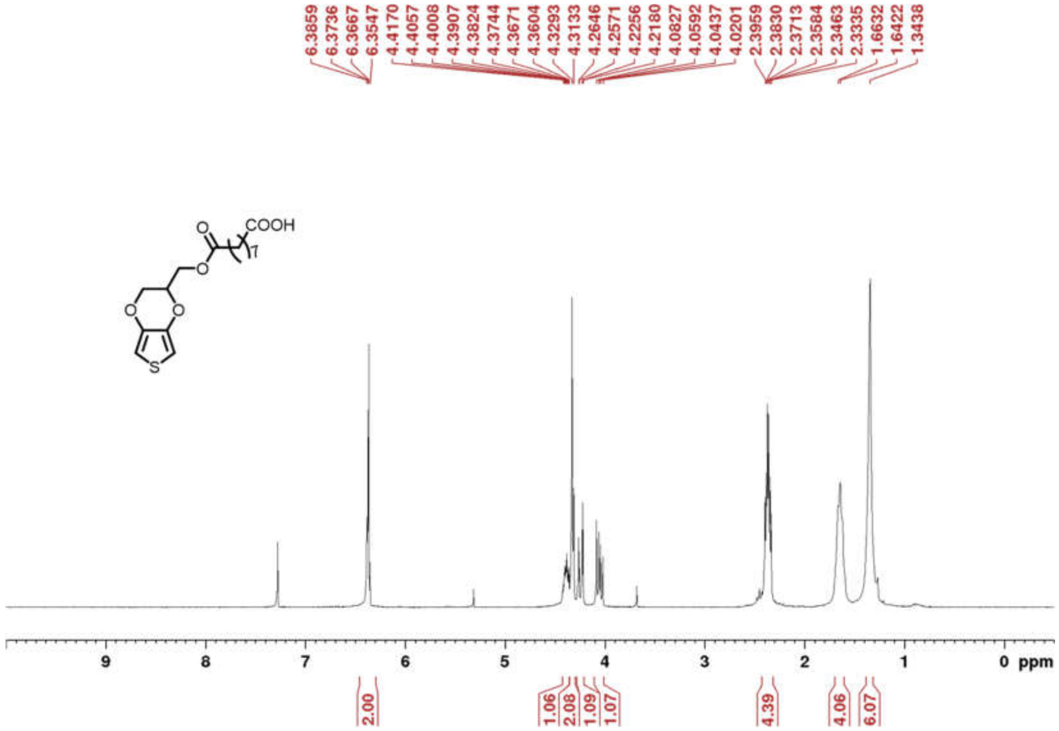


**Figure S8.** $^{1}$H NMR spectrum of C$_9$-EDOT-COOH (300 MHz, CDCl$_3$)

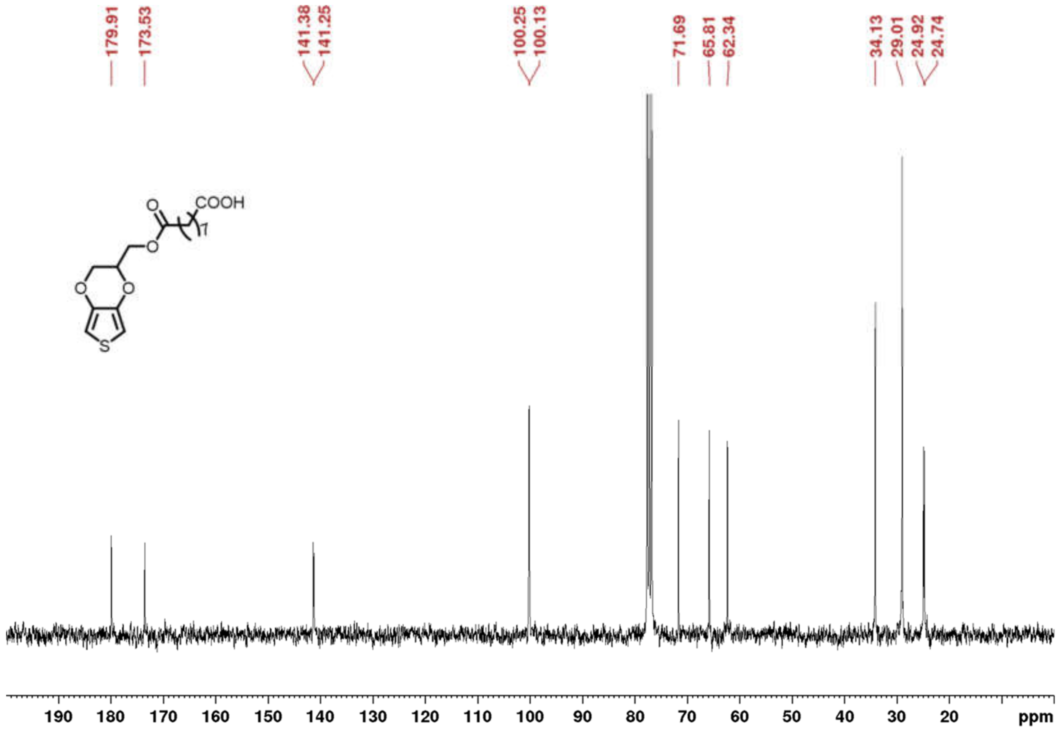


**Figure S9.** $^{13}$C NMR spectrum of C$_9$-EDOT-OOH (75 MHz, CDCl$_3$)

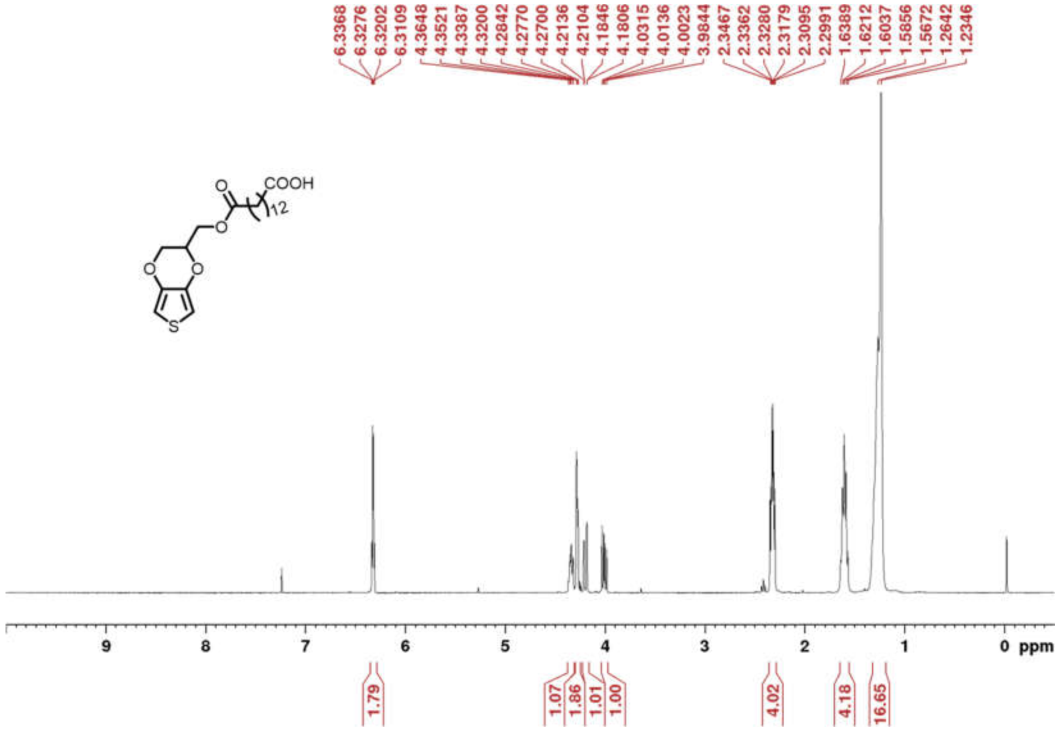


**Figure S10.** $^{1}$H NMR spectrum of $C_{14}$-EDOT-COOH (400 MHz, $CDCl_3$)

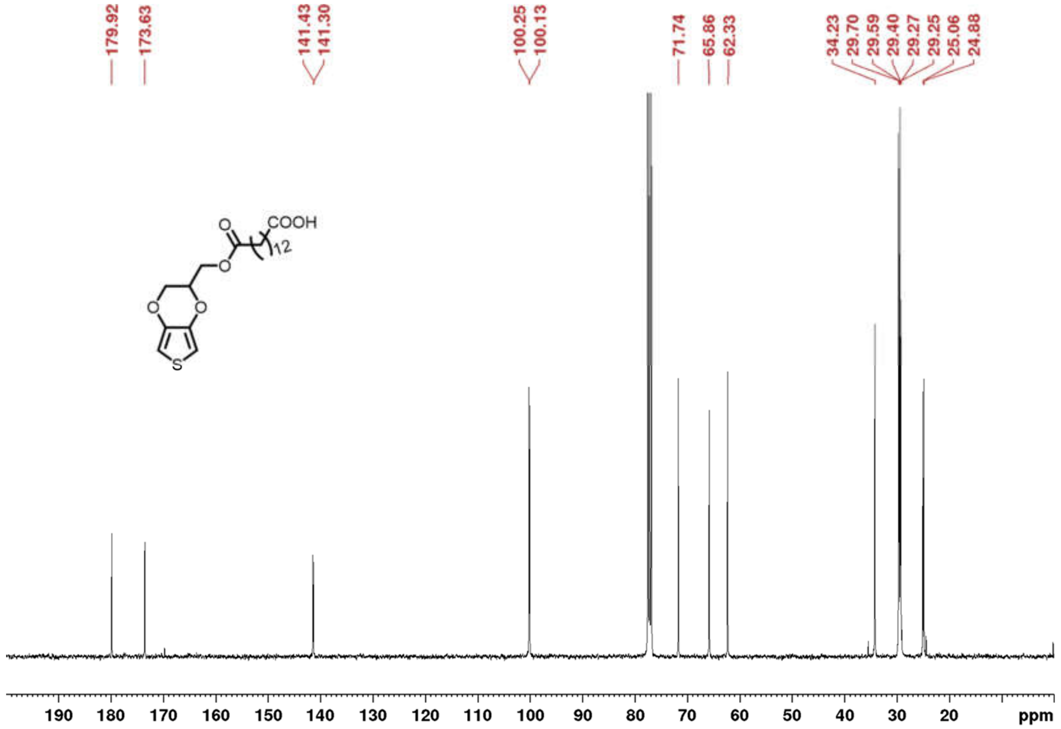


**Figure S11.** $^{13}$C NMR spectrum of $C_{14}$-EDOT-COOH (100 MHz, $CDCl_3$)

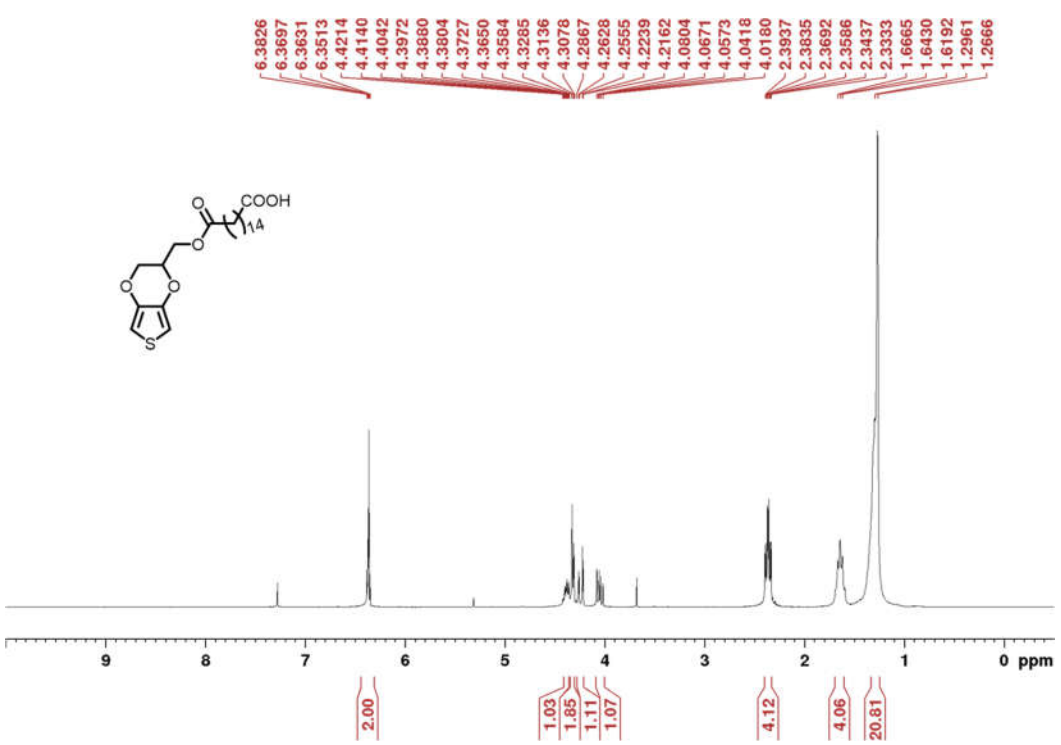


**Figure S12.** $^{1}H$ NMR spectrum of $C_{16}$-EDOT-COOH (300 MHz, $CDCl_3$)

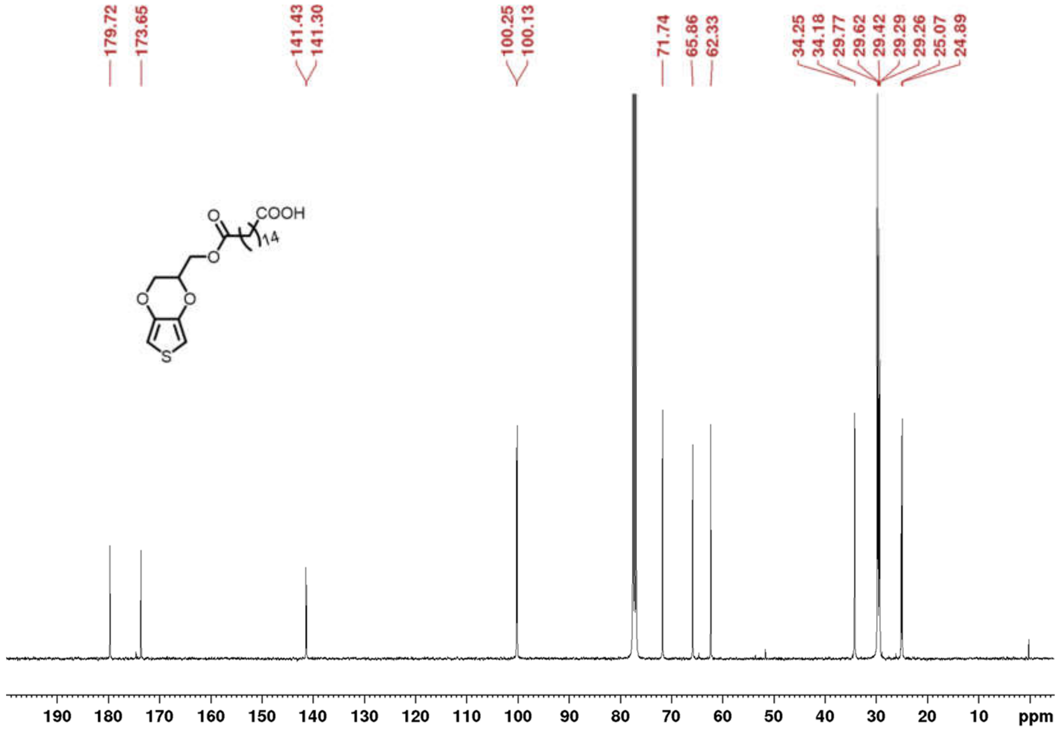


**Figure S13.** $^{13}C$ NMR spectrum of $C_{16}$-EDOT-COOH (100 MHz, $CDCl_3$)

**Mass Spectra of $C_n$-EDOT-COOH monomers**

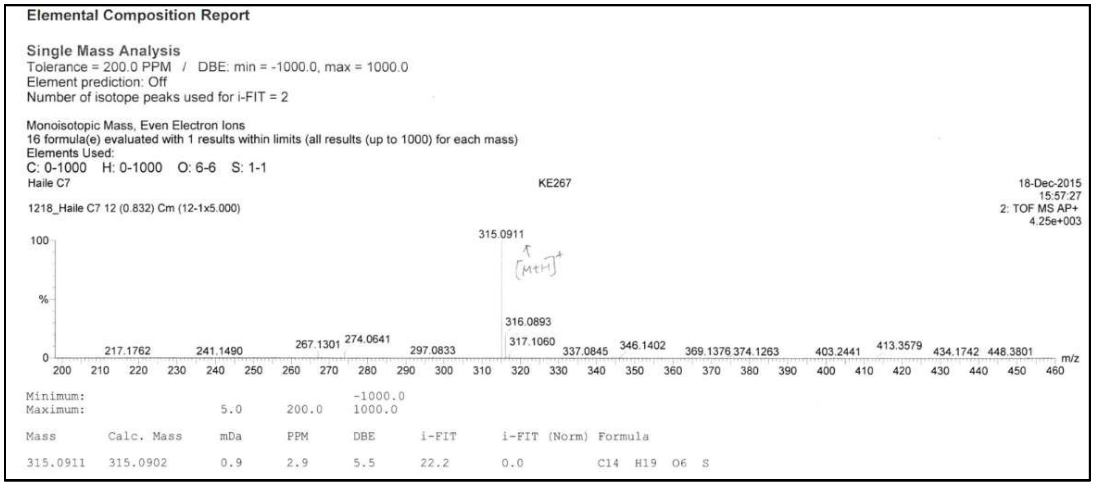


**Figure S14.** MS of $C_7$-EDOT-COOH

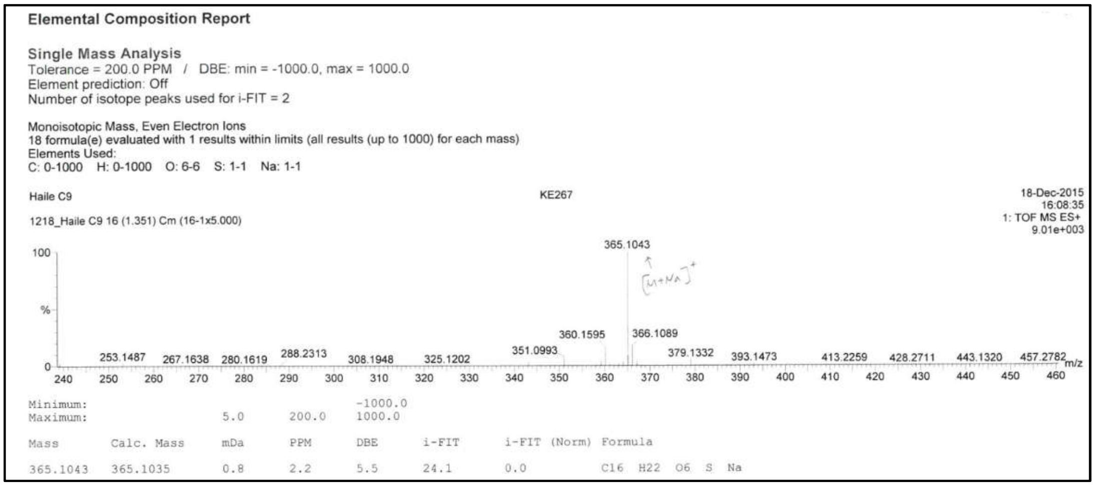


**Figure S15.** MS of $C_9$-EDOT-COOH

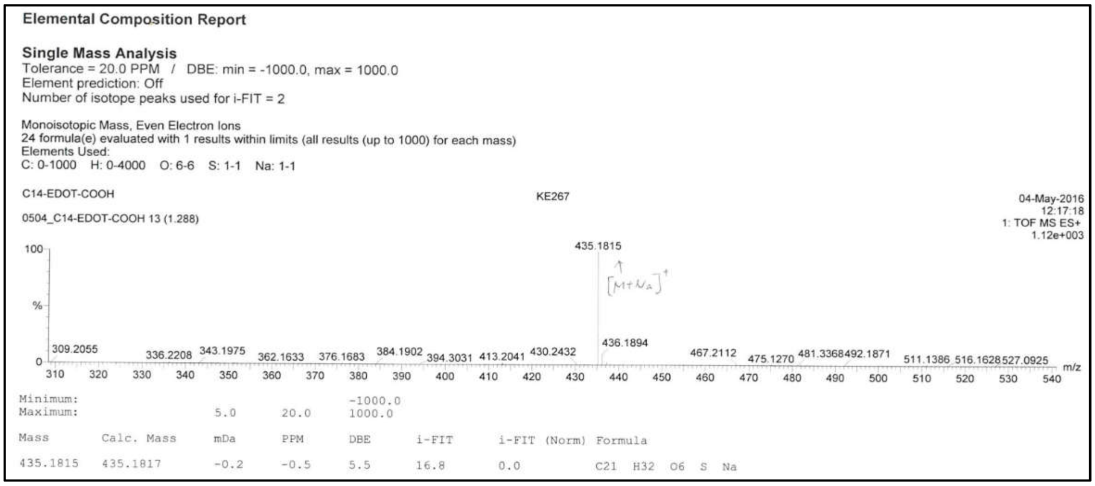


**Figure S16.** MS of $C_{14}$-EDOT-COOH

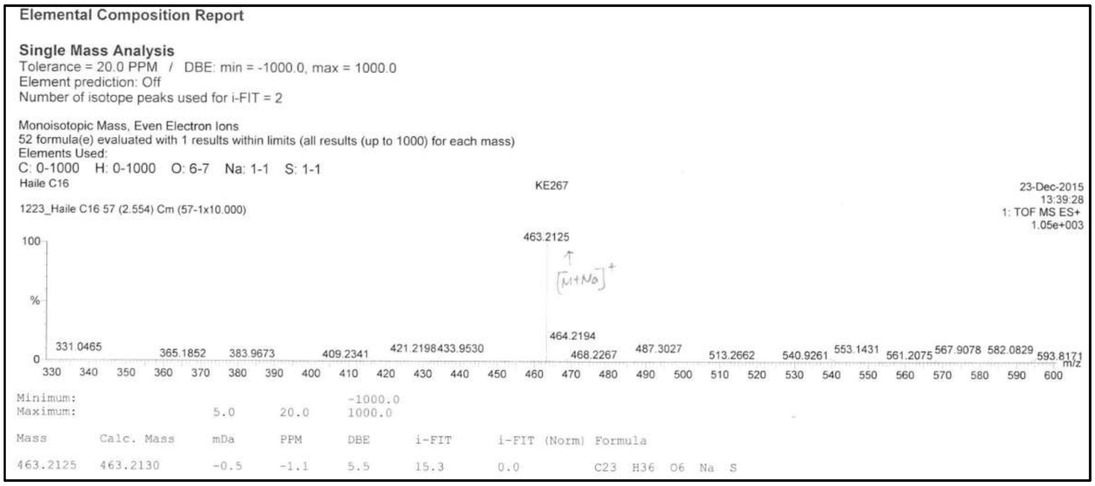


**Figure S17.** MS of $C_{16}$-EDOT-COOH
